# Assessing the quality of Hand Hygiene data produced by Alberta Health Services using a time-in-motion study

**DOI:** 10.1017/ash.2024.247

**Published:** 2024-09-16

**Authors:** Jennifer Ellison, Zhe Lu, Alison Devine, Blair McFerran, Sandra Bolton, Helen Popson, Kathryn Bush

**Affiliations:** Infection Prevention & Control, Alberta Health Services; Alberta Health Services

## Abstract

**Background:** Alberta Health Services (AHS) measures hand hygiene compliance through direct observations performed by trained site-based reviewers (SBRs) and facilitated by the Infection Prevention and Control (IPC) program. Within AHS there are >100 acute care facilities, ranging in bed size from four beds to more than 1,000, with catchment populations ranging from one million. A time-in-motion study using trained AHS IPC staff was proposed to validate the completeness and accuracy of data being collected by the SBRs. **Methods:** The AHS IPC staff performed direct observations at pre-selected facilities across all five zones and four different unit types (emergency, medical, surgical, and intensive care) for four 30-minute periods during weekdays between June and September 2023. An iPad app was used to capture results from all four moments of hand hygiene. The reviewer indicated the day and time of the review and captured as many representative hand hygiene moments and healthcare providers as possible. The distributions of the four moments of hand hygiene, healthcare provider group and overall compliance were compared at the unit type and facility level (tertiary, large urban, regional, pediatric, and small sites) between this time-in-motion study and SBR data collected June-September 2023. **Results:** The study collected 175 reviews and 4,683 observations from 14 facilities and 48 units. Between June and September 2023, SBRs collected 2,625 reviews and 61,506 observations from these same facility and unit types. Across all facility and unit types, the distribution of the four moments was similar between the study and SBRs. Similar proportions of healthcare providers were also observed. However, the overall hand hygiene compliance collected in the study was approximately 10% lower across all unit types as compared to that collected by the SBRs (study: 63%-84%; SBRs: 75%-92%). **Conclusions:** In public health surveillance, completeness and accuracy are two characteristics of high-quality data. A time-in-motion study identified that the hand hygiene observations collected by SBRs were complete, as the range of healthcare providers observed, and the distribution of their moments, mirrored that collected in the study. However, the SBRs reported higher compliance than the study participants and the true hand hygiene compliance is likely lower than what is currently being reported. Since this difference was seen consistently across all unit and facility types, trending data over time should still identify areas in need of improvement and may help to suggest causes of the over-reporting.

